# More than just mild thrombocytopenia: Clinical clues in the diagnosis of germline predisposition to malignancy from a rare *ETV6* variant

**DOI:** 10.1002/ccr3.7273

**Published:** 2023-07-02

**Authors:** Shannon Fang, Juliana Perez Botero, Lisa R. Hackney

**Affiliations:** ^1^ Case Western Reserve University School of Medicine Cleveland Ohio USA; ^2^ Versiti Diagnostic Laboratories Milwaukee Wisconsin USA; ^3^ Division of Hematology and Oncology Medical College of Wisconsin Milwaukee Wisconsin USA; ^4^ Pediatric Hematology and Oncology UH Rainbow Babies and Children's Hospital Cleveland Ohio USA

**Keywords:** genetics, hematology, oncology, pediatrics and adolescent medicine, thrombocytopenia

## Abstract

**Key Clinical Message:**

In the evaluation of patients with longstanding mild thrombocytopenia, emphasis on family history, genetic testing, and collaborative clinical and laboratory‐based family studies can ensure proper diagnosis and monitoring for malignancies.

**Abstract:**

We report the diagnostic approach to mild and non‐specific thrombocytopenia with unclear genetic findings in two sisters. Genetic sequencing revealed a rare variant in ETS Variant Transcription Factor 6, which is associated with inherited thrombocytopenia with predisposition to hematologic malignancy. Familial studies provided sufficient evidence for a likely pathogenic classification.

## Graphical abstract

Familial testing reveals a rare *ETV6* variant likely causing inherited thrombocytopenia in this family.

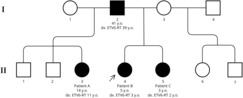

## 1 INTRODUCTION

Thrombocytopenia can be the first indication of an underlying condition in children and includes a wide range of differential diagnoses. Inherited thrombocytopenias are a heterogenous group of disorders with low platelet counts, with or without platelet dysfunction. With increasing access to genetic testing, the number of genes associated with this phenotype and number of patients diagnosed continue to grow.^1^ Over 50 genes have been described to be associated with inherited thrombocytopenia and an estimated 2.7% are from disease‐causing variants in ETS Variant Transcription Factor 6 (*ETV6*).^1^, ^2^ ETV6 is a transcription repressor in the ETS family of transcription factors, and it is implicated in normal hematopoiesis, thrombopoiesis, and platelet function.^3^, ^4^ Distinguishing *ETV6*‐related thrombocytopenia (*ETV6*‐RT) from other etiologies is important, given its associated risk of malignancy,^2^, ^3^, ^4^ and requires genetic testing for diagnostic confirmation. This report describes the diagnostic approach and clinical challenges in a family with inherited thrombocytopenia that was found to carry a rare disease‐causing *ETV6* variant.

## 2 CASE DESCRIPTION

### 2.1 Patient A

An 11‐year‐old female patient was referred to the pediatric hematology clinic for isolated thrombocytopenia, which was identified after presenting to the emergency department with bilateral toe bruising. Her fingers and toes would become purple for a prolonged period of time, but bleeding symptoms were absent.

Family history was positive for bruising in the mother and anemia in the maternal grandmother. The patient was taking atomoxetine, risperidone, hydroxyzine, and medroxyprogesterone acetate. Physical examination showed cyanosis in the feet after exposure to cold, slow capillary refill, and macular papular red erythematous rashes above the ankles bilaterally.

Complete blood count (CBC), screening coagulation studies, serum immunoglobulins, and autoimmune studies were obtained (Table 1). Mild thrombocytopenia with normal sized platelets and normal granulation on peripheral smear was noted. The clinical presentation was most consistent with Raynaud's, and it was thought that the mildly decreased platelets could have been the result of viral suppression or side effects from risperidone. A repeat CBC 1 month later was recommended.

**TABLE 1:** Laboratory values in members of a family with the *ETV6* c.1085A>G variant.

	Patient A	Patient B	Patient C	Father	Reference
Platelets	**133**	**105**	**137**	**138**	150–400 × 10^9^/L
WBC	7.7	5.5	7.4	11.2	4.5–13.5 × 10^9^/L
Hemoglobin	15.3	**11.1**	**11.2**	15.6	12.0–16.0 g/dL
Hematocrit	42.6	**31.1**	**32.9**	45.3	36.0%–46.0%
MCV	88	89	88	97	78–102 fL
ANC	4.69	2.36	2.68	6.75	1.20–7.70 × 10^9^/L
Absolute lymphocyte	2.33	3.14	3.58	2.54	1.80–5.00 × 10^9^/L

*Note*: Abnormal values are in bold. All units are in accordance with the reference values.

Abbreviations: ANA, antinuclear antibody; ANC, absolute neutrophil count; MCV, mean corpuscular volume; WBC, white blood cell.

### 2.2 Patient B

A 3‐year‐old female patient with a past medical history of chronic urticaria and reactive airway disease was referred to hematology by allergy and immunology due to low platelet count. She had no history of infection, fever, or sick exposures. She reported easy bruising when active and presented with bilateral leg bruising. CBC results were similar to patient A (Table 1).

Family history revealed Patient B is the half‐sister of Patient A, both of whom share a father. The father and three of his children have a history of low platelet counts. No other family members were symptomatic or needed treatment for their thrombocytopenia.

While the differential diagnosis for isolated mild thrombocytopenia is broad, family history pointed towards an inherited etiology. Von Willebrand factor (VWF) plasma studies were normal. Platelet transmission electron microscopy (PTEM) showed ultrastructurally normal platelets, with some platelets having large and decreased alpha granules. Bone marrow biopsy/aspirate had normal maturing trilineage hematopoiesis and showed no evidence of leukemia. There were reduced megakaryocytes and few hypolobated forms.

### 2.3 Genetic testing

**FIGURE 1:**
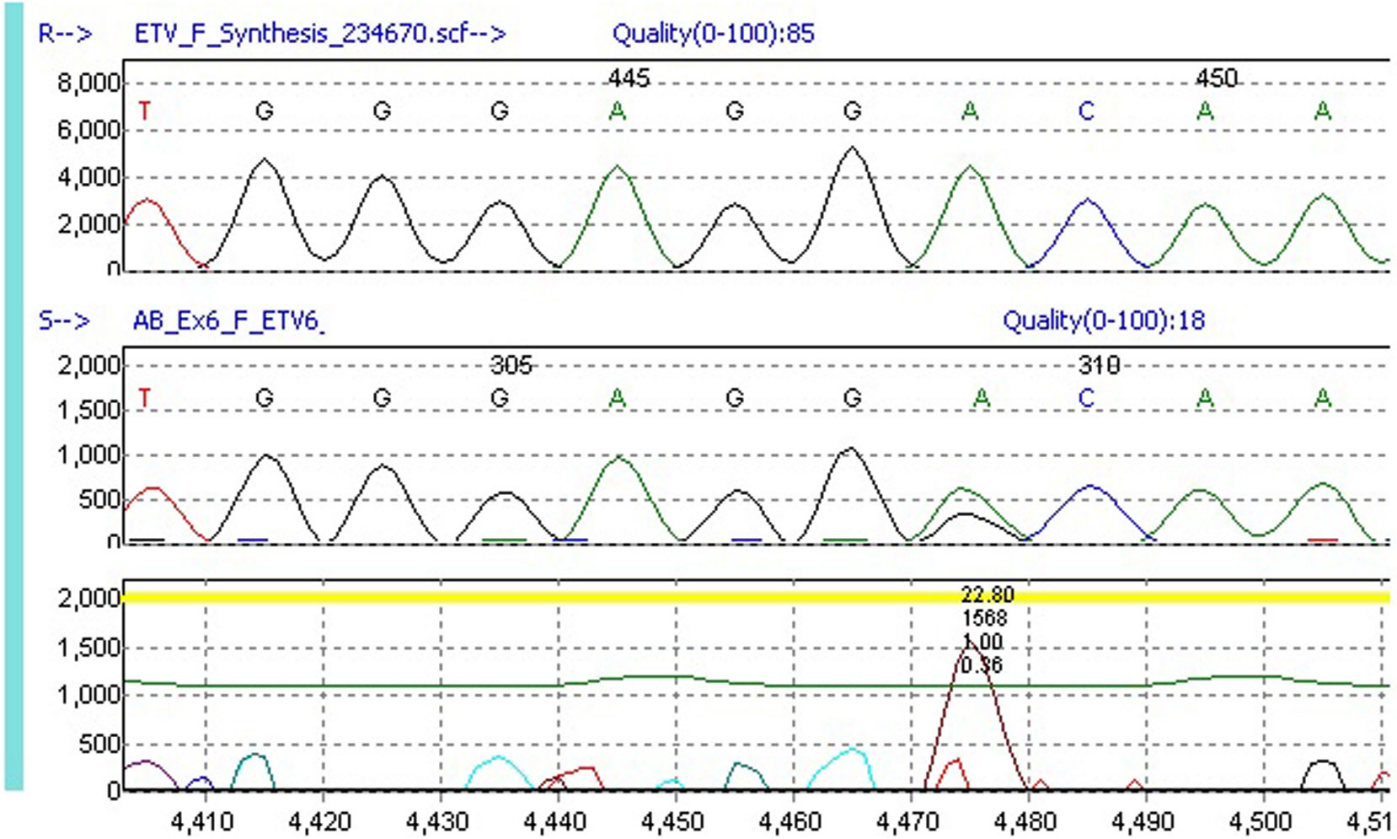
Sanger sequencing confirming the heterozygous *ETV6* c.1085A>G variant analyzed by Mutation Surveyor. The upper panel shows reference sequence; the middle panel shows patient sequence; the lower panel shows the variant as a peak.

A next‐generation sequencing (NGS) based panel of genes associated with inherited thrombocytopenia was performed at Versiti Diagnostic Laboratories in a blood sample of patient B. The germline heterozygous *ETV6* c.1085A>G (p.Asp362Gly) missense variant was identified (Figure 1). This variant occurs in a well‐conserved nucleotide and is located in the ETS domain, the functional domain in ETV6 that binds DNA. The variant had not been reported in the literature or in the general population. Due to limited evidence, using the criteria developed by the American College of Medical Genetics (ACMG) and the Association for Molecular Pathology (AMP), the variant was classified as a variant of uncertain significance (VUS) (PM2, PP3, PP4). However, it was suspected to be implicated in the low platelet counts seen in this family.

**FIGURE 2:**
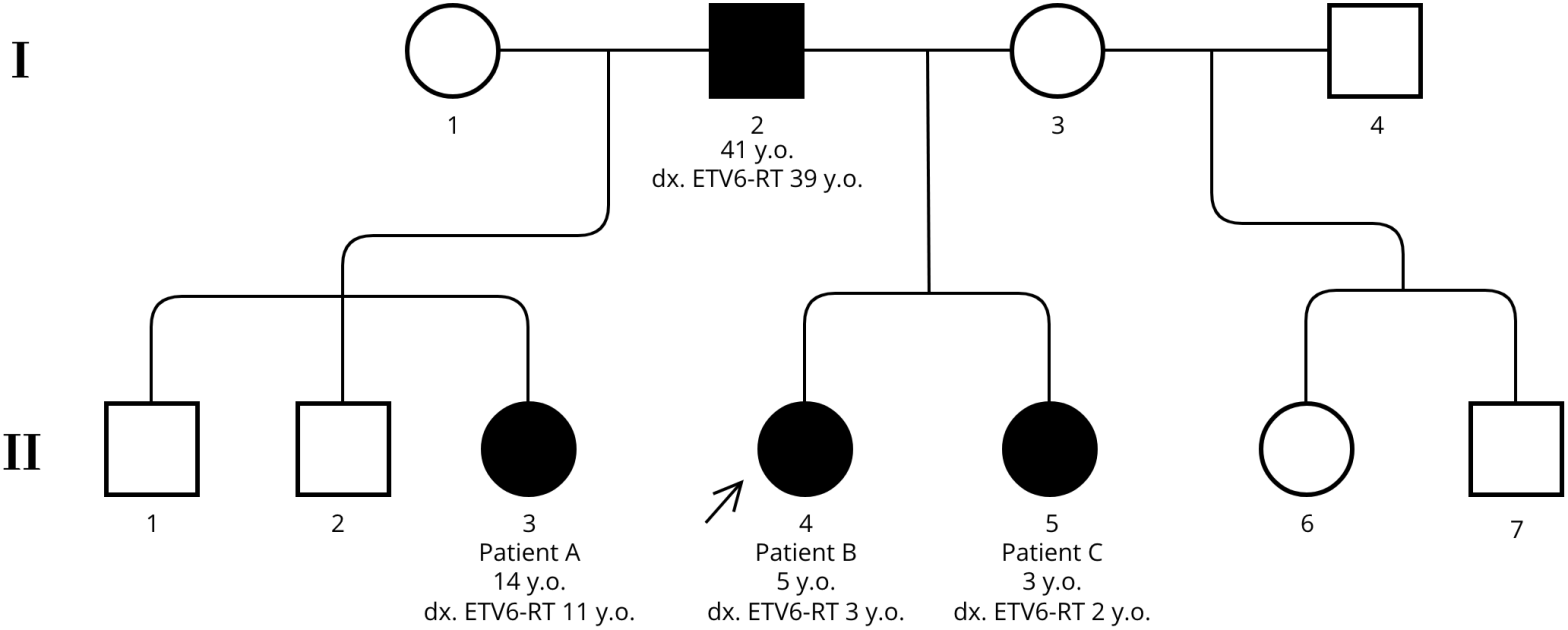
Pedigree showing the segregation of the *ETV6* c.1085A>G variant within the extended family of the proband (patient B). WT, wild type gene confirmed by genetic testing. Arrow indicates proband.

Familial testing to provide additional evidence for variant classification using segregation was undertaken (Figure 2). The variant segregated with the thrombocytopenic trait in all individuals tested. Those who were *ETV6* wild type had normal platelet counts. Subtle elevations in red blood cell size were noted and considered part of the collective additional evidence. Familial studies provided sufficient evidence to reach a likely pathogenic classification for the familial variant (PM2, PP1_mod, PP4, PP3).

## 3 DISCUSSION

*ETV6*‐RT, as illustrated by the study of this family, presents with mild and variable clinical manifestations that can easily be overlooked, especially when historical platelet counts and a complete family history are not readily available. Typical platelet counts in patients with *ETV6*‐RT are in the mild to moderate thrombocytopenia range, usually >75 × 10^9^/L.^4^, ^5^ Platelet size is normal, and platelet morphology or peripheral smear findings do not show distinguishing features that set it apart from other inherited thrombocytopenias.^3^, ^5^ Mean corpuscular volume can be mildly elevated in patients with *ETV6*‐RT,^4^, ^6^ and while not specific to this disorder, is a useful clinical clue when present. Bleeding is often variable; impaired platelet aggregation with adenosine diphosphate and arachidonic acid, as well as abnormal alpha granule morphology on PTEM, have been described.^6^ Patients with *ETV6‐RT* can exhibit megakaryocytes that are small and hypolobulated,^4^, ^6^, ^7^ as in the case of patient B. The inheritance pattern is autosomal dominant, and the penetrance of the thrombocytopenic phenotype is high. The family's history showed affected family members in multiple generations, with males and females affected equally, supporting this inheritance pattern.

When the clinical evaluation of a patient and their family is suggestive of an inherited thrombocytopenia without pathognomonic clinical features, genetic testing becomes an essential tool in confirming the diagnosis and providing appropriate genetic counseling. At least three nonsyndromic autosomal dominant inherited thrombocytopenias with normal platelet size are associated with hematologic malignancy and involve variants in *RUNX1*, *ANKRD26,* and *ETV6*.^8^, ^9^, ^10^ Genetic testing is the only strategy able to confidently distinguish them from each other, which is an important step in the clinical approach because the expected rate of evolution to malignancy and type of associated neoplasm varies between them. *ETV6*‐RT has an overall 30% risk for hematologic malignancies—including B‐cell acute lymphoblastic leukemia, acute myeloid leukemia, and myelodysplastic syndrome.^4^

Results of genetic testing can be difficult to interpret, especially in cases where the identified variant is rare or novel. In absence of prior reports in the literature or functional evidence to support its pathogenicity, other evidences—including thorough knowledge of the functional domains of the protein affected, population data, and in silico prediction tools—are helpful in variant classification. However, due to insufficient evidence, frequently the classification is VUS, which is not clinically actionable.^11^ As illustrated in this family, segregation can provide key additional evidence for variant classification and resolution of the VUS.

Inherited thrombocytopenias with mild decrease in platelet counts and no syndromic associations, such as *ETV6*‐RT, are challenging to diagnose due to their non‐specific clinical presentation and low frequency compared to acquired platelet disorders. Recognizing the clinical clues of inherited thrombocytopenia is an important diagnostic skill to prevent misdiagnosis and ensure proper counseling regarding risk of malignancy. Unexplained, persistent thrombocytopenia should prompt consideration of inherited syndromes, particularly with a positive family history, and appropriate genetic counseling and testing. Special attention to family history, genetic testing, and family studies using multidisciplinary clinical and laboratory‐based teams can facilitate confirmation of an inherited thrombocytopenia diagnosis, even when limited variant‐specific data are available.

## AUTHOR CONTRIBUTIONS

**Shannon Fang:** Conceptualization; visualization; writing – original draft; writing – review and editing. **Juliana Perez Botero:** Conceptualization; data curation; formal analysis; investigation; writing – original draft; writing – review and editing. **Lisa Hackney:** Conceptualization; data curation; formal analysis; funding acquisition; investigation; writing – original draft; writing – review and editing.

## FUNDING INFORMATION

Funding was received from University Hospitals Rainbow Babies & Children's Department of Pediatric Hematology Oncology.

## CONFLICT OF INTEREST STATEMENT

The authors have no conflicts of interest to declare.

## CONSENT

Written informed consent was obtained from the patient to publish this report in accordance with the journal's patient consent policy. Patient consent was obtained.

## Data Availability

Data sharing is not applicable to this article as no new data were created or analyzed in this study.
